# Impairment of Fas-ligand–caveolin-1 interaction inhibits Fas-ligand translocation to rafts and Fas-ligand-induced cell death

**DOI:** 10.1038/s41419-017-0109-1

**Published:** 2018-01-22

**Authors:** Xenia A. Glukhova, Julia A. Trizna, Olga V. Proussakova, Vladimir Gogvadze, Igor P. Beletsky

**Affiliations:** 1 Institute of Theoretical and Experimental Biophysics, Russian Academy of Sciences, Puschino, Russia 142290; 2 Faculty of Fundamental Medicine, MV Lomonosov Moscow State University, Moscow, Russia 119991; 3 Division of Toxicology, Institute of Environmental Medicine, Karolinska Institutet, Box 210, 17177 Stockholm, Sweden

## Abstract

Fas-ligand/CD178 belongs to the TNF family proteins and can induce apoptosis through death receptor Fas/CD95. The important requirement for Fas-ligand-dependent cell death induction is its localization to rafts, cholesterol- and sphingolipid-enriched micro-domains of membrane, involved in regulation of different signaling complexes. Here, we demonstrate that Fas-ligand physically associates with caveolin-1, the main protein component of rafts. Experiments with cells overexpressing Fas-ligand revealed a FasL N-terminal pre-prolin-rich region, which is essential for the association with caveolin-1. We found that the N-terminal domain of Fas-ligand bears two caveolin-binding sites. The first caveolin-binding site binds the N-terminal domain of caveolin-1, whereas the second one appears to interact with the C-terminal domain of caveolin-1. The deletion of both caveolin-binding sites in Fas-ligand impairs its distribution between cellular membranes, and attenuates a Fas-ligand-induced cytotoxicity. These results demonstrate that the interaction of Fas-ligand and caveolin-1 represents a molecular basis for Fas-ligand translocation to rafts, and the subsequent induction of Fas-ligand-dependent cell death. A possibility of a similar association between other TNF family members and caveolin-1 is discussed.

## Introduction

Fas-ligand (FasL), a member of the tumor necrosis factor (TNF) family, is a type II protein, which can exist in both transmembrane and secreted (soluble) forms^1,2^. FasL consists of extracellular, transmembrane, and intracellular domains. The extracellular part is responsible for the recognition of relevant receptors, Fas-antigen and DcR3, and self-association of the ligand^3,4^. Тhe transmembrane region of FasL is less studied; apparently, it is responsible for anchoring this molecule to the plasma membrane. The intracellular part of FasL is essential for sorting into secretory lysosomes, the translocation of the ligand into lipid rafts, and also for the reverse signal transduction into cells bearing transmembrane FasL^5–10^. FasL localization, reverse signaling, and its expression are mediated by the interaction of intracellular domains of FasL and other intracellular proteins. Nowadays, several proteins have been identified which can be associated with FasL, specifically with prolin-rich domains (PRD), able to bind peptide-containing Src homology3 (SH3) or WW-domain-binding sites^11–14^.

The sequence analysis shows that the intracellular part of FasL contains a putative caveolin-1-binding motif, Y_7_PYPQIYW_14_. Caveolin-1, a 22-kDa integral membrane protein, is a major protein component of caveolae, a special type of lipid rafts^15,16^. These structures were detected in the plasma membrane, endosomes, and Golgi apparatus; they participate in endocytosis, transcytosis, and intracellular signal transduction^17,18^. Сaveolin-1 can regulate raft-dependent cellular processes indirectly or directly. In the former case, for instance, caveolin-1 can recruit cholesterol to raft domains and modulate membrane lipid composition, affecting endocytosis of lipid rafts^19–22^. The direct effect of caveolin-1 on raft-dependent functions is caused by its ability to interact with a variety of proteins, including different signaling molecules^22,23^. Many, but not all, caveolin-1-interacting proteins contain one of the two related caveolin-1-binding motifs (ΦXΦXXXXΦ or ΦXXXXΦXXΦ, where Φ is an aromatic amino acid). These motifs mediate the interaction of caveolin-1-binding proteins with the scaffolding domain of caveolin-1^24–27^. Given that the ability of FasL to induce cell death requires its localization to rafts^8,9^, a possible interaction of caveolin-1 with FasL is of special interest.

In this study, we investigated the direct physical interaction between FasL and caveolin-1, and attempted to identify respective binding domains. Our data demonstrate that FasL interacts directly with caveolin-1. The FasL intracellular region contains two caveolin-binding sites and deletion of both of these disrupts the routing of FasL to lipid rafts, and makes cells more resistant to Fas-mediated cell death. These results suggest that caveolin-1 may regulate FasL location and, respectively, modulate FasL-dependent cell death.

## Results

### Overexpression of FasL causes its translocation into rafts and cell death

**Fig. 1:**
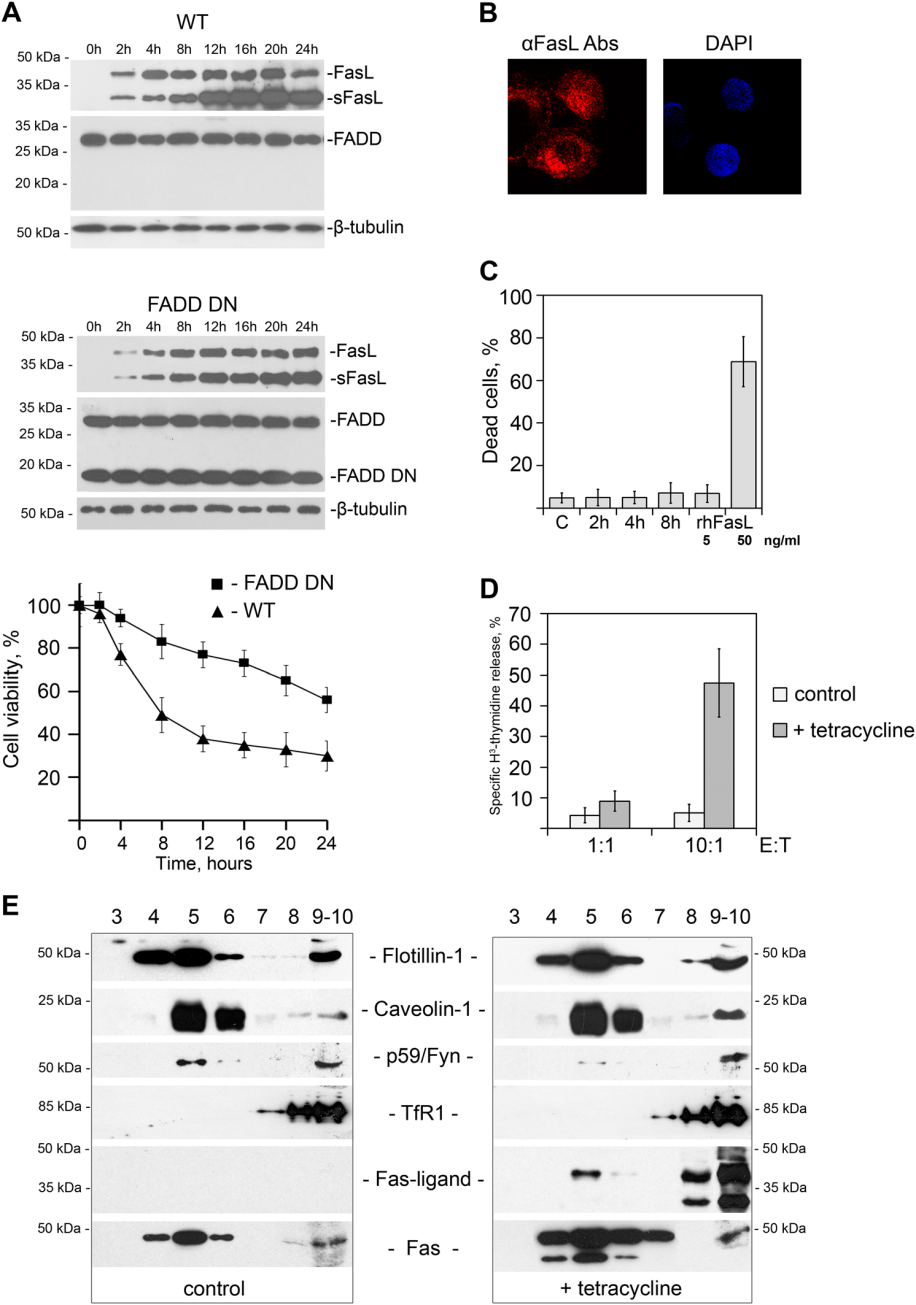
Overexpression of FasL causes its translocation into rafts and induces cell death **a** Immunoblot analysis of FasL, FADD DN, and ß-tubulin expression in tetracycline-treated HeLa-pcDNA4/TO-FasL and HeLa-pcDNA4/TO-FasL–FADD DN cells in the specified time periods. *FasL* full-length Fas-ligand, *sFasL*short form of FasL^45^, *FADD* Fas-associated death domain protein, *FADD* *DN* a dominant negative FADD mutant protein. β-tubulin is used as a loading control. Survival of HeLa cells overexpressing FasL alone (WT) (▲) and FasL together with the dominant negative FADD/MORT1 (FADD DN) (■), incubated with tetracycline for various time intervals (chart). Cell viability was determined by the neutral-red uptake method. Results are presented as means of at least three separate experiments, where error bars represent s.e.m. of biological triplicates. **b** HeLa-pcDNA4/TO-FasL cells were fixed and processed for immunolabeling for FasL (red) following by staining with the Alexa Fluor® 610-R-phycoerythrin goat anti-mouse Abs or DAPI (blue). **c** Survival of U937 cells incubated with HeLa-pcDNA4/TO-FasL condition medium collected 2, 4, and 8 h after tetracycline induction or 5 and 50 ng/ml of rhFasL (recombinant human FasL). Cell viability was determined via an MTT assay. **d** 3H-thymidine-labeled U937 cells were co-incubated with HeLa-pcDNA4/TO-FasL cells at various E:T ratios in the presence or absence of tetracycline. The percent of specific 3H-thymidine release from the target cells was determined after 12 h incubation. Results are presented as means of at least three separate experiments, where error bars represent SD of biological triplicates. **e** Lysates of non-induced (left panel) and tetracycline-induced for 4 h HeLa-pcDNA4/TO-FasL cells (right panel) were separated using ultracentrifugation in sucrose gradient. The distribution of raft-specific (Flotillin-1, Caveolin-1, p59Fyn) and non-specific (TfR1) markers as well as FasL and Fas was analyzed by immunoblotting

Normally, the level of FasL expression in proliferating cells is low, since its enhanced expression can lead to apoptosis in Fas-bearing cells. In order to reach a high level of FasL, we produced HeLa cells with tetracycline-inducible FasL expression (HeLa-pcDNA4/TO-FasL), and showed that overexpression of FasL caused cell death, which was blocked by dominant negative FADD/MORT1 (Fig. 1a)^28^. Hence, the cell death was triggered by the Fas-mediated signaling rather than the “reverse” apoptotic signaling via transmembrane FasL^29^. Immunostaining of cells with antibodies to FasL has revealed that overexpressed FasL is localized to cytosol and the plasma membrane, but not to the nucleus (Fig. 1b).

It has been shown previously that the transmembrane and secreted forms of FasL can trigger the death of cells expressing Fas^30^. Using U937 cells bearing Fas and that die upon treatment with FasL, we checked the cytotoxicity of the medium in which HeLa-pcDNA4/TO-FasL cells were cultured, for 2, 4, and 8 h after tetracycline induction. As can be seen in Fig. 1c, none of the obtained supernatant samples containing short form of FasL (sFasL) up to 8 ng/ml at 12 h was able to kill cells, whereas recombinant soluble FasL (50 ng/ml) killed almost all cells within 12 h. Co-cultivation of control and tetracycline-treated HeLa-pcDNA4/TO-FasL cells, together with U937 cells labeled with H^3^-thymidine at the ratio 10:1, demonstrated excessive U937 cell death within 12 h (Fig. 1d). Thus, one can assume that the transmembrane, but not the secreted, FasL causes the death of both HeLa-pcDNA4/TO-FasL and U937 cells. Subsequent experiments were performed using only HeLa-pcDNA4/TO cells transfected with various forms of FasL.

It has been shown previously that the translocation of FasL into rafts is essential for cell death^8,9^. In order to test FasL distribution after the stimulation of its expression, we used gradient centrifugation^31^. After 4 h of induction, FasL was found in the raft fraction together with traditional raft markers such as flotillin-1, caveolin-1, and Fyn-kinase (Fig. 1e)^32,33^. Analysis of the transferrin receptor distribution, used as a negative control, showed that this protein is localized exclusively in non-raft fractions. Thus, overexpression of FasL in Hela cells results in its translocation into rafts and subsequent cell death.

### FasL is physically associated with caveolin-1

**Fig. 2:**
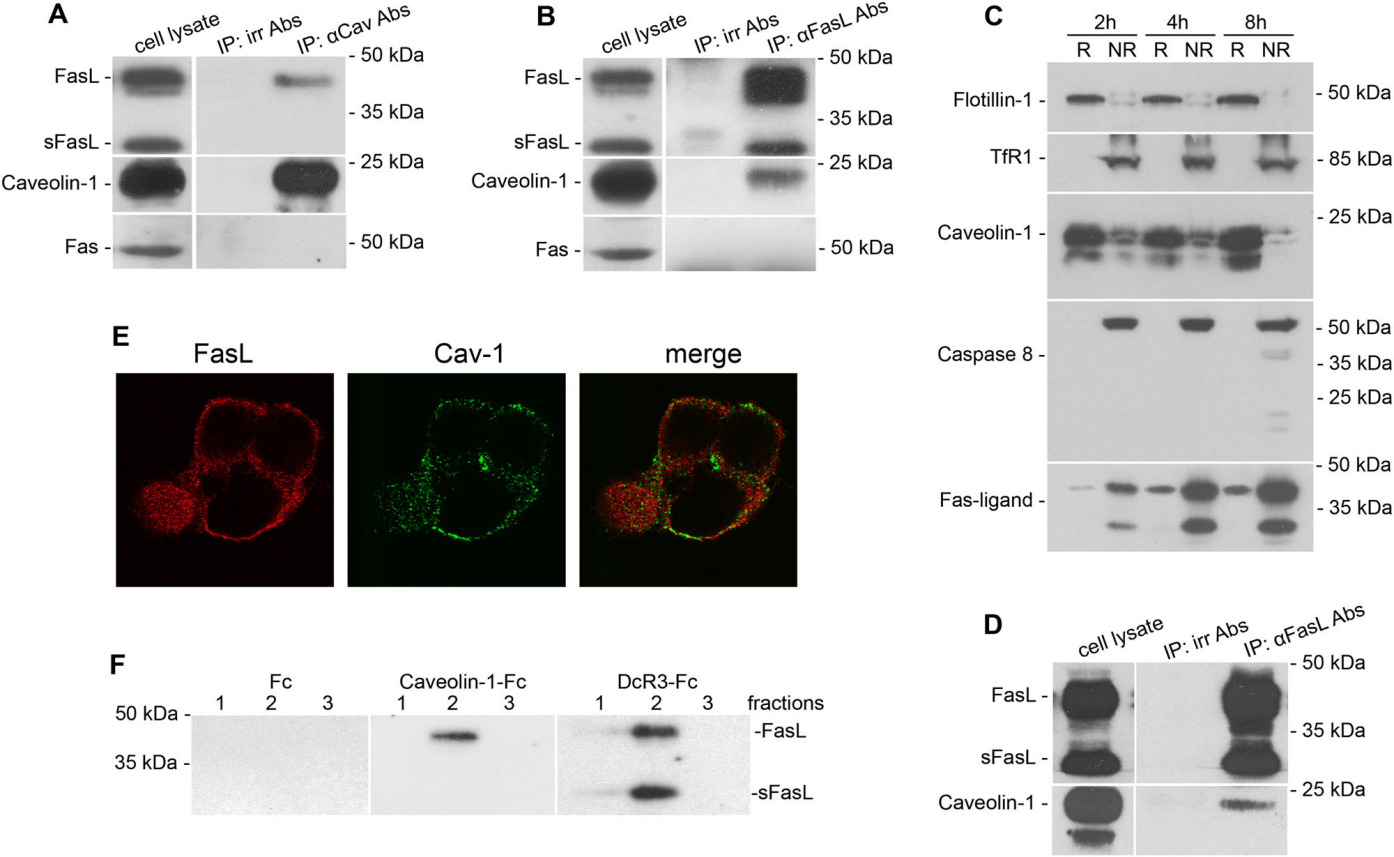
FasL physically interacts with caveolin-1 Lysates of HeLa-pcDNA4/TO-FasL cells induced by tetracycline for 4 h were immunoprecipitated with caveolin-1 Abs (**a**) or anti-Fas-ligand mAbs (**b**). Precipitates were analyzed by immunoblotting and stained with anti-Fas-ligand mAbs (**a**) or anti-caveolin-1 Abs (**b**), respectively. Both precipitates were analyzed by immunoblotting and stained with anti-Fas mAbs as well (lower panels of **a** and **b**). Irrelevant Abs were used as a negative control. **c** Immunoblot analysis of caveolin-1, caspase-8, and FasL expression in raft (R) and non-raft fractions (NR) of HeLa-pcDNA4/TO-FasL–FADD DN cells. Raft and non-raft fractions were prepared from lysates of tetracycline-induced for 2, 4, and 8 h HeLa-pcDNA4/TO-FasL–FADD DN cells. Flotillin-1 and TfR1 were used as raft-specific and non-specific markers, respectively. **d** Lysates of HeLa-pcDNA4/TO-FasL–FADD DN cells induced by tetracycline for 4 h were immunoprecipitated with anti-Fas-ligand mAbs followed by immunoblotting and staining with anti-caveolin-1 Abs. **e** Co-localization of FasL and caveolin-1. HeLa-pcDNA4/TO-FasL cells were fixed and processed for double immunolabeling for caveolin-1 (green) and FasL (red) followed by staining with the fluorescein goat anti-rabbit IgG and Alexa Fluor® 610-R-phycoerythrin goat anti-mouse Abs, respectively. **f** Interaction between recombinant caveolin-1 and FasL. Resins containing recombinant caveolin-1- Fc, DcR3-Fc (positive control), and Fc fragment of human Ig (negative control) were incubated with the lysate of tetracycline-induced HeLa-pcDNA4/TO FasL cells. Bound proteins were eluted and subjected to immunoblotting using anti-Fas-ligand mAbs

Caveolin-1 is a major protein in lipid rafts^15,16^, taking part in intracellular signal transduction, particularly in cell-death signaling^34^. To test a possible physical association between FasL and caveolin-1, lysates of cells overexpressing FasL (HeLa-pcDNA4/TO-FasL) were immunoprecipitated with an anti-caveolin-1 antibody. Anti-caveolin-1 immunoprecipitation followed by FasL staining revealed the co-precipitation of FasL and caveolin-1 (Fig. 2a). In turn, anti-FasL antibodies co-precipitated a small amount of caveolin-1 (Fig. 2b). Similar data were obtained after immunoprecipitation of the lysates of cells overexpressing FasL и dominant negative FADD/MORT1 (HeLa-pcDNA4/TO-FasL–FADD DN) (Fig. 2c, d). It should be mentioned that in these cells, caspase-8 activation was observable only 8 h after tetracycline induction, whereas the presence of FasL in rafts and the association between caveolin-1 and FasL were observed after 2 and 4 h, respectively (Fig. 2c, d). Thus, the association is not a consequence of procaspase-8 cleavage and cell-death activation.

Double immunostaining of cells with antibodies to FasL and caveolin-1 also demonstrated the partial co-localization of FasL and caveolin-1 (Fig. 2e).

The association between caveolin-1 and FasL was confirmed in the experiments with recombinant fusion protein caveolin-1-Fc (where Fc is a fragment of human IgG1). As a positive control, DcR3-Fc protein, which binds FasL, was used; as a negative control—Fc fragment. Cell lysates were incubated with resins to which recombinant proteins were bound covalently. After washing, proteins captured from lysates were eluted and stained with anti-FasL antibody. FasL was found in elutes from resins bearing DcR3-Fc and caveolin-1-Fc, but not the Fc fragment alone (Fig. 2f). It should be mentioned that in precipitates, the short form of FasL was not detected (Fig. 2a and f), which indicates that sFasL is not involved in the interaction with caveolin-1.

It has been shown earlier that Fas-antigen can interact physically with caveolin-1^35^. Based on our findings, one can assume that FasL–caveolin-1 association can be mediated by Fas-antigen. However, we could not detect Fas-antigen in immunoprecipitates obtained (Fig. 2a and b). These results confirm that FasL and caveolin-1 can interact directly, without the participation of the Fas-antigen. Further experiments with recombinant FasL and caveolin-1 confirmed this conclusion.

### N-terminal domain of FasL is essential for its association with caveolin-1, raft translocation, and cell-death activation

**Fig. 3:**
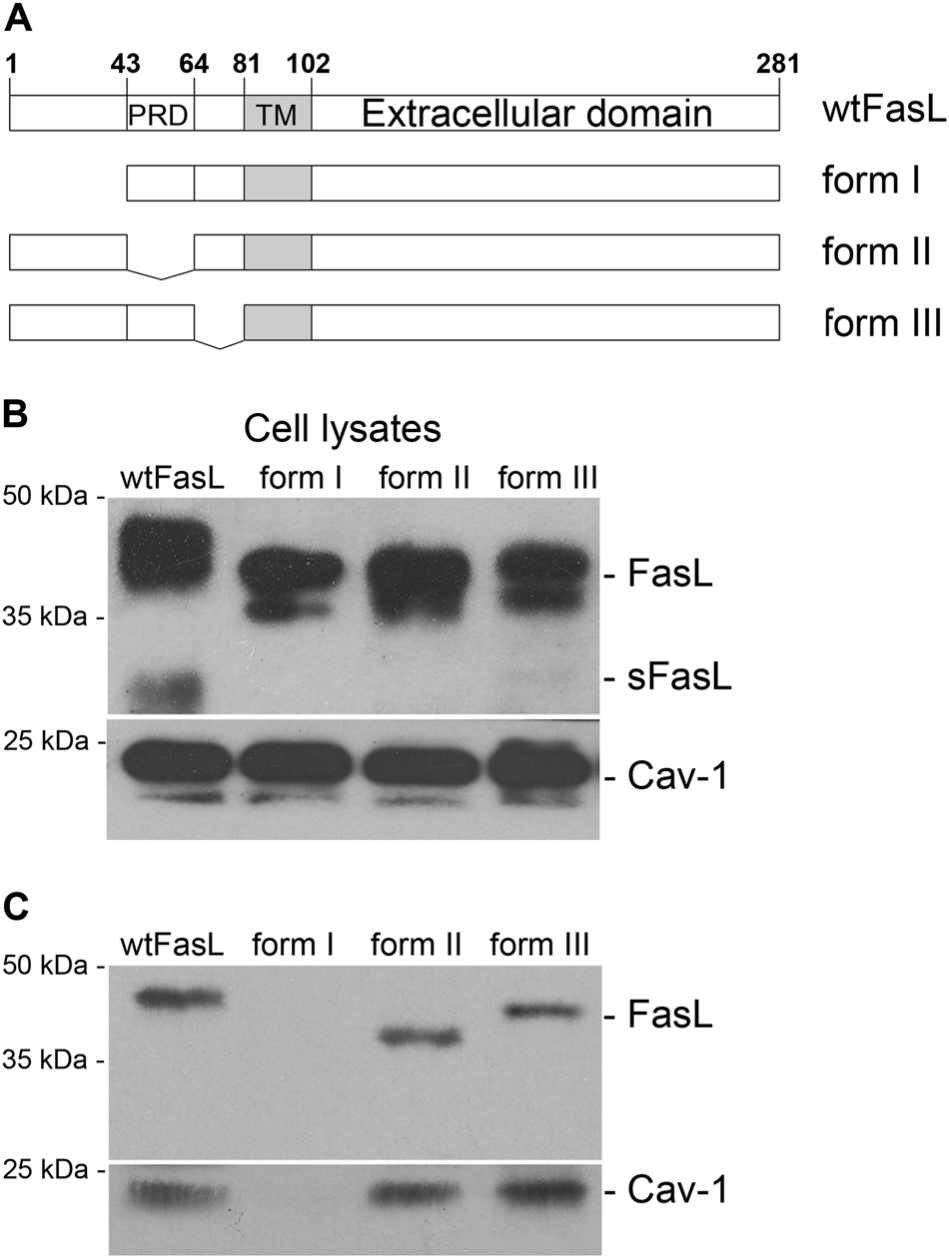
N-terminal domain of FasL is important for interaction with caveolin-1 **a** Schematic diagram of FasL and its mutant forms. *PRD* polyproline domain, *TM* transmembrane domain. **b** Immunoblot analysis of FasL and caveolin-1 expression in HeLa cells transfected with wild (wt) and mutant forms of FasL (form I: Δ1–42, form II: Δ43–63, and form III: Δ64–80). **c** HeLa cells overexpressing wild type (wt) and mutant forms (Δ1–42, Δ43–63, and Δ64–80) of FasL were lysed, and immunoprecipitation with anti-caveolin-1 Abs was carried out followed by staining with anti-Fas-ligand mAbs (upper panel), or lysates were immunoprecipitated with anti-Fas-ligand mAbs followed by staining with anti-caveolin-1 Abs (lower panel)

Three domains with different binding properties can be recognized in the intracellular part of the FasL-PRD (43–63 aa), the N-terminal pre-PRD region (1–42 aa), and the C-terminal post-PRD (64–80 aa). It has been shown previously that the N-terminal region (1–40 aa) and the PRD of FasL are necessary for raft recruitment and the regulation of FasL activity^8,9^. In order to identify the region(s) within the FasL intracellular part, which interacts directly with caveolin-1, we prepared several transfected cell lines expressing inducible mutant forms of FasL with deletions at 1–42 аа (without N-terminal domain, form I), 43–63 аа (without PRD, form II), and 64–80 aa (without C-terminal, form III) (Fig. 3a). In the selected clones, the levels of expression of caveolin-1 and mutant and full-length forms of FasL were almost the same (Fig. 3b). Immunoprecipitation of various FasL mutants demonstrated that unlike forms II, III, or full-length FasL, form I did not interact with caveolin-1 (Fig. 3c).

**Fig. 4:**
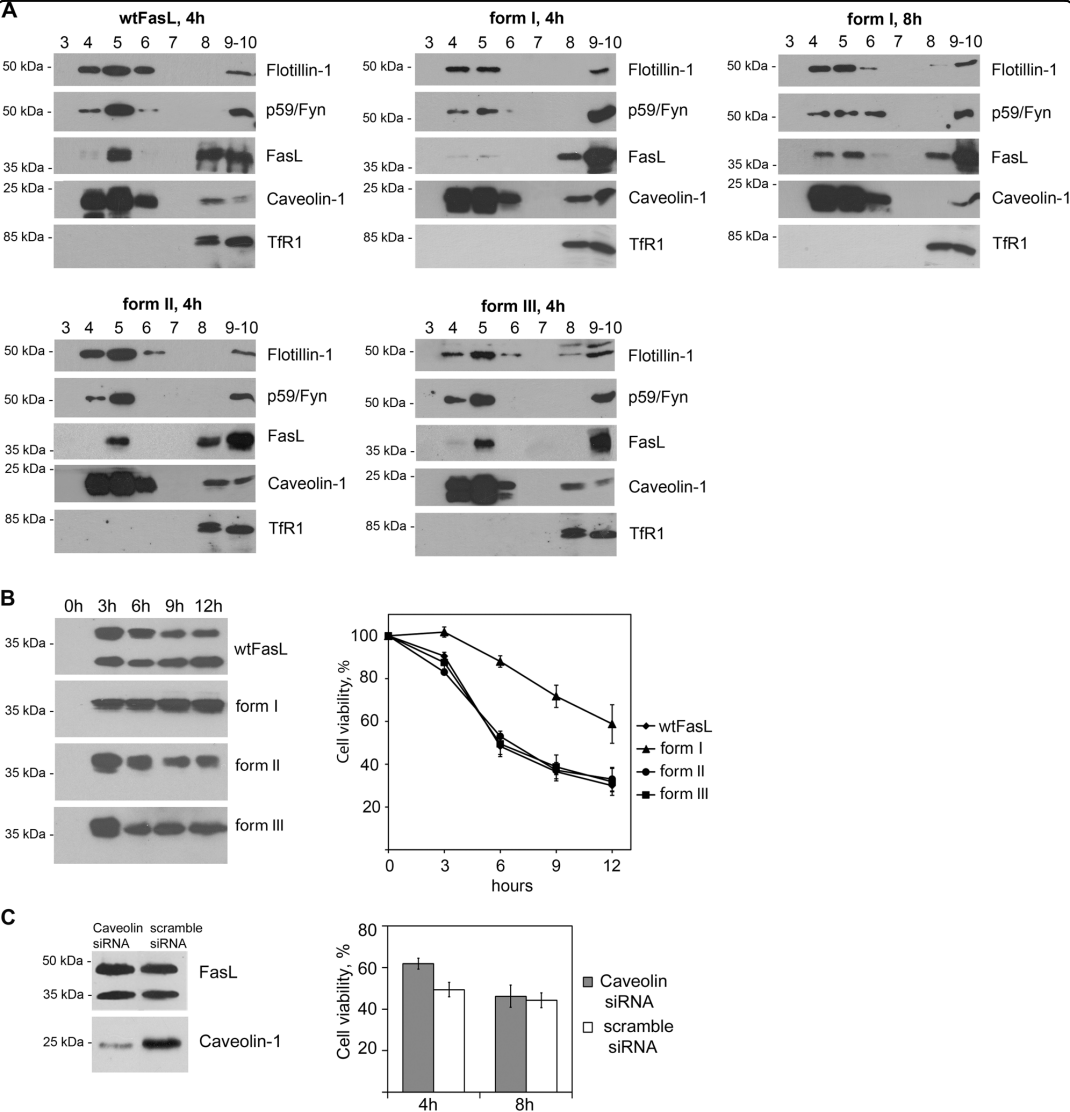
N-terminal domain of FasL is essential for its translocation to raft and cell-death activation **a** The distribution of wild-type and mutant forms of FasL between raft (4–6) and non-raft (9,10) fractions was assessed using corresponding antibodies. **b** Expression levels of FasL and its mutant forms in the specified time periods were estimated by immunoblot analysis with anti-FasL Abs. Survival of HeLa cells overexpressing FasL variants, incubated with tetracycline during the indicated intervals (chart). Cell viability was determined by the neutral-red uptake assay. Results are presented as mean ± s.e.m of three independent experiments (each in triplicate). **c** Caveolin-1 knockdown by siRNA in HeLa-pcDNA4/TO-FasL cells decreases FasL cytotoxic effect. HeLa-pcDNA4/TO-FasL cells were transfected with psiRNA-h7SK-caveolin or psiRNA-h7SK-scramble control. Cell lysates were stained with anti-FasL mAbs (upper panel) and anti-caveolin-1 Abs (lower panel). Survival of HeLa-pcDNA4/TO-FasL cells transfected with psiRNA-h7SK-caveolin or psiRNA-h7SK-scramble control (chart). Cell viability was determined by the neutral-red uptake method. Results are presented as mean ± s.e.m. of four experiments (each in triplicate)

Next, we tested if the differences in binding to caveolin-1 can affect FasL distribution and FasL-mediated cell death. Density gradient fractionation of detergent lysates revealed that in contrast to forms II, III, and the wild-type FasL found in rafts 4 h after tetracycline induction, together with traditional raft markers such as flotillin-1, caveolin-1, and Fyn-kinase^36,37^, form I appeared only after 8 h (Fig. 4a). Analysis of transferrin receptor 1 distribution used as a negative control showed that this protein was localized exclusively in non-raft fractions. Interestingly, cells expressing form I were more resistant to FasL-mediated apoptosis, whereas cells expressing forms II and III died similarly to cells bearing full-length FasL (Fig. 4b).

Indirect confirmation of the role of caveolin-1 in FasL-dependent cell death came from the caveolin-1 knockout experiments. The caveolin-1 gene was knocked out in HeLa-pcDNA4/TO-FasL cells, using siRNA technique. We were not able to downregulate caveolin-1 completely. However, even in these cells, FasL-dependent death was less pronounced compared to cells with a normal content of caveolin-1 (Fig. 4c).

These results show that FasL PRD and C-terminal post-PRD are not involved in caveolin-1 binding, whereas the FasL N-terminal pre-PRD is essential for the association with caveolin-1, translocation to lipid rafts, and cell-death activation.

### N-terminal pre-PRD of FasL contains two caveolin-binding sites

**Fig. 5:**
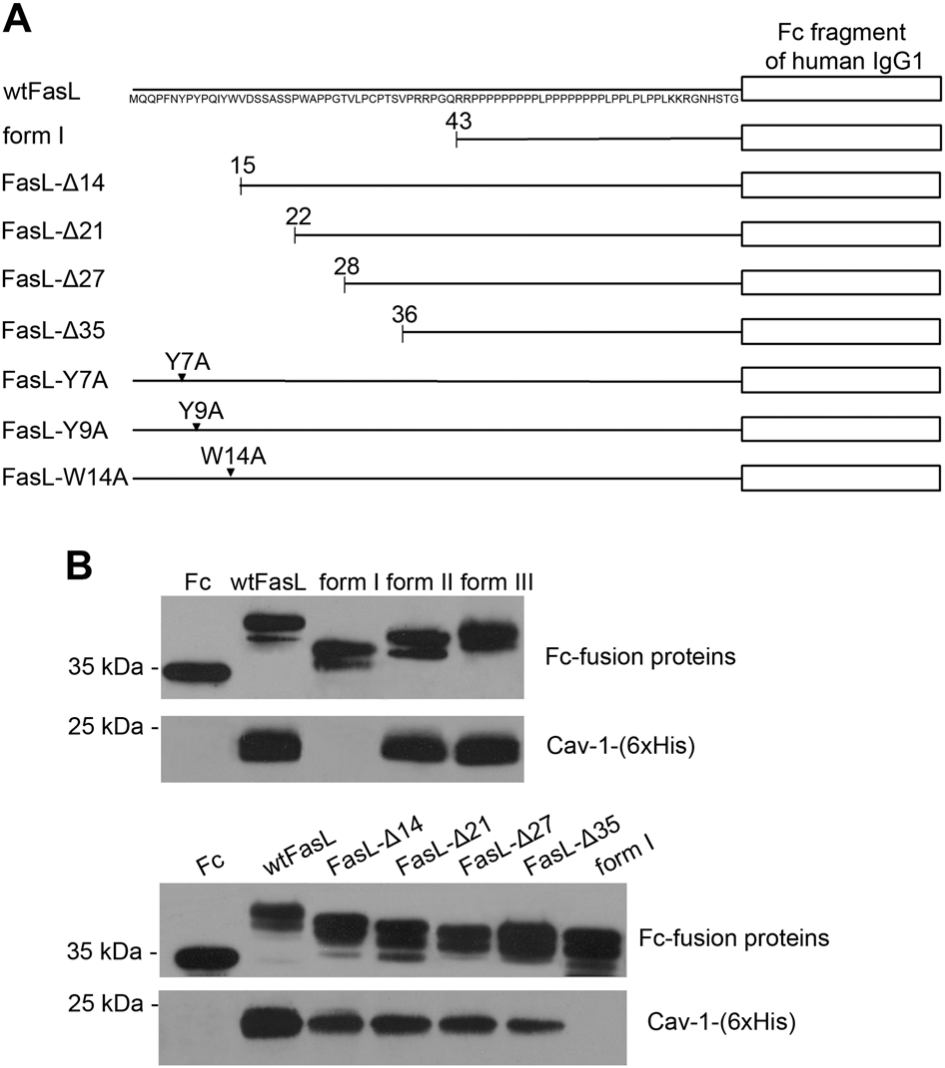
Mapping of FasL domains responsible for the interaction with caveolin-1 **a** Schematic diagram showing various deletion mutants of FasL N-terminal pre-PRD fusion protein **b** Recombinant caveolin-1 (6xHis) was incubated with wild-type (FasL) or FasL mutant intracellular domain - Fc fusion proteins. Associates were stained with anti-human Fc Abs (upper panels) and anti-caveolin-1 Abs (lower panels)

Next, the region(s) within the N-terminal domain of FasL, which are responsible for the binding to caveolin-1, were defined. Recombinant fusion proteins of the Fc fragment of human IgG1 (Fc) and various deletion mutants of the N-terminal domain of FasL were used to identify specific site(s) of binding of caveolin-1-(6xHis) (Fig. 5a). An in vitro binding assay followed by immunoblotting revealed that the FasL N-terminal domain lacking the first 14 amino acids (FasL-Δ14), including the putative caveolin-1 binding motifs, exhibited a drastic decrease in binding (about fivefold) as compared to the full-length N-terminal domain (Fig. 5b). Additional deletion of the FasL N-terminal domain up to the 35th amino acid (Ser 35, FasL-Δ35) did not cause any further alteration in the interaction as compared to FasL-Δ14. However, even the FasL N-terminal domain lacking the first 35 amino acids (FasL-Δ35) was still able to bind to caveolin-1-(6xHis) (Table 1). This allows us to conclude that the N-terminal domain of FasL bears two caveolin-binding sites: within first 14 amino acids (FasL-Δ14) and between 36 and 42 amino acids (FasL-Δ35).

**Table 1:** Binding of the FasL intracellular domain mutants with the full-length caveolin-1 and its N- or C-terminal domains

	Cav-1-(6xHis)	Caveolin 1–101 aa	Caveolin 135–178 aa
wtFasL	+	+	+
Form I	–	−	−
FasL-Δ14	+	−	+
FasL-Δ21	+	−	+
FasL-Δ27	+	−	+
FasL-Δ35	+	−	+
FasL-Y7A	+	−	+
FasL-Y9A	+	−	+
FasL-W14A	+	−	+

To further test if these interactions mediated by two sites might differ depending on the various domains of caveolin-1, we carried out a set of experiments with N- or C-terminal domains of caveolin-1 (caveolin 1–101 aa and caveolin 135–178 aa, respectively) fused with glutation-S-transferase (GST)^38^. The N-terminal domain of caveolin-1 interacted with the FasL full-length N-terminal domain only. Moreover, the replacement of single aromatic amino acids within the first caveolin-binding site (Y7, Y9, and W14) for alanine prevented binding to the N-terminal domain of caveolin-1. The C-terminal domain of caveolin-1 was able to bind all the FasL deletion mutants with a similar affinity as the full-length caveolin-1, except for form I (1–42 аа deletion) (Table 1). Therefore, the N-terminal domain of caveolin-1 is responsible for the binding of the caveolin-binding site within the first 14 amino acids of FasL, whereas the C-terminal domains of caveolin-1 appear to take part in the interaction with the second caveolin-binding site (36–42 amino acids of FasL).

### Localization in heavy detergent-resistant membranes (DRM-H)

**Fig. 6:**
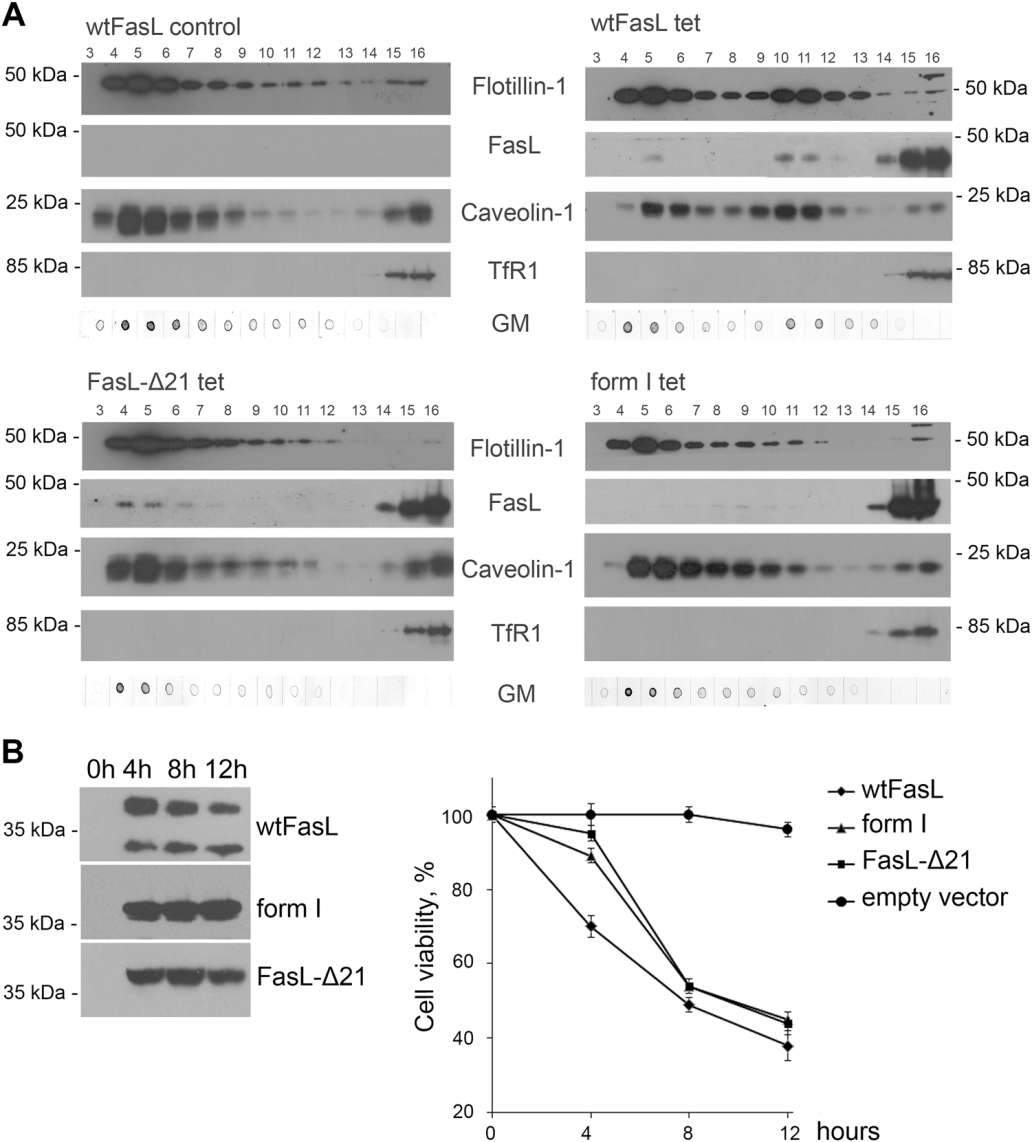
Deletion of caveolin-binding sites in N-terminal domain results in Fas-ligand mislocalization and cell-death suppression **a** Lysates of non-induced (left upper panel), tetracycline-induced for 4 h HeLa-pcDNA4/TO-FasL cells (right upper panel), HeLa cells transfected with mutant forms of FasL-Δ21 (left lower panel), and form I (right lower panel) were separated by ultracentrifugation in a sucrose gradient. Distribution of raft-specific (flotillin-1, caveolin-1, GM1 ganglioside (GM)) and non-specific (transferrin receptor1 (TfR1)) markers as well as FasL was analyzed by immunoblotting. **b** Expression levels of FasL and its mutant forms in the specified time periods were estimated by immunoblot analysis with anti-FasL Abs. Survival of HeLa cells overexpressing wild-type and mutant forms (FasL-Δ21 and form I) of FasL, incubated with tetracycline for the times indicated (chart). Cell viability was determined by the neutral-red uptake assay. Results are presented as mean ± s.e.m. of four experiments (each in triplicate)

FasL was found in lipid rafts, and it has been shown that removal of the intracellular domain diminishes its transport into rafts (herein and refs.^9,39^). The observed association of FasL with caveolin-1 described above suggests that cellular localization of FasL can be affected by caveolin-1. To prove this hypothesis, we investigated the distribution of FasL mutants in detergent-resistant membrane (DRM) micro-domains. For these experiments, the standard protocol^31^ was modified; in particular, the lysates were subjected to centrifugation in a sucrose gradient (5%/20%/35%/40%) at 38,000 rpm for 20 h. Including an additional 20% sucrose solution allowed the separation of DRM into two subpopulations (fractions 4–6 и 10–12). DRMs were isolated from control cells, cells transfected with wild-type FasL, and cells bearing deletions of one (FasL-Δ21) or both caveolin-binding sites (form I). Fractions obtained were stained for FasL, caveolin-1, markers for rafts (GM1 ganglioside, flotillin-1), and, as a negative control, transferrin receptor 1 (Fig. 6a).

In cells with full-length FasL, as well as FasL-Δ21 and form I cells, raft proteins caveolin-1 and flotillin were mainly co-localized between 5% and 20% sucrose in fractions 4–6, which corresponds to classical DRM localization, and also at the bottom (non-raft fractions). After overexpression of FasL, a part of caveolin-1 appeared in fractions 10–12 (the border between 20% and 35% sucrose), which was probably due to the formation of complexes between caveolin-1 and FasL. However, in cells with mutated FasL (FasL-Δ21 и form I), caveolin-1 was only found in fractions 4–6, similar to the control cells. In the case of wild-type and FasL-Δ21, FasL was co-localized with caveolin-1; however, the deletion of the first 42 amino acids in FasL disturbed its distribution between various DRM subpopulations.

Membranes insoluble in Triton X-100 localized between 20% and 35% sucrose are supposed to be enriched with proteins, and therefore to have higher buoyant density as compared to classical rafts. Presumably they form upon raft binding to adaptor or cytoskeleton proteins, and participate in signaling or transport processes^40,41^. Thus, one can conclude that the first 42 amino acids of FasL participating in FasL–caveolin-1 complex formation are responsible for ligand translocation into DRM, wherein 21 amino acids are apparently able to form large protein complexes, which besides caveolin might include other unidentified molecules.

The importance of FasL–caveolin-1 association within DRM is strengthened by cytotoxic tests in cell cultures transfected by FasL and its mutant forms (Fig. 6b). Deletion of one or both caveolin-1-binding sites causes a substantial decrease in FasL cytotoxicity.

## Discussion

Molecular mechanisms controlling the targeting FasL and other members of TNF ligand family into rafts, as well as those regulating signal direction, “forward” or “reverse”, remain poorly understood. That is why the intracellular part of FasL, which can bind a variety of different proteins, remains the main object of investigations^11–14^. In the present work, we showed for the first time that FasL can interact with caveolin-1. Using mutant forms of FasL, we uncovered two caveolin-binding sites, within the first 14 amino acids (FasL-Δ14) and between 36 and 42 amino acids, in the N-terminal domain of FasL. In addition, one can suggest that the presence of a casein kinase I motif recognition site, S_17_SASS_21_, characteristic of many members of the TNF ligand family^42^, in close proximity to a caveolin-1 binding motif, might also influence the formation of the FasL–caveolin-1 complex.

**Fig. 7:**
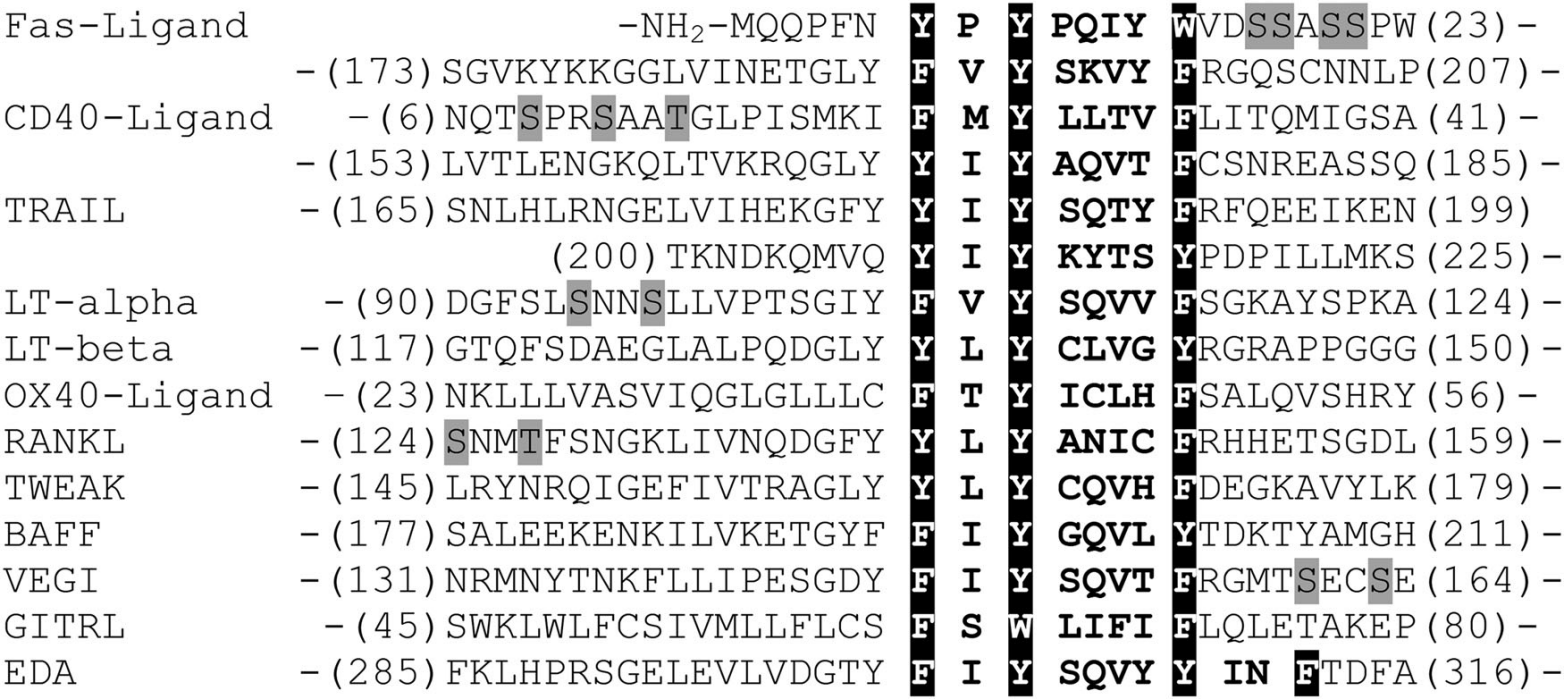
Comparison of putative caveolin-binding sites within 11 members of the TNF family Putative caveolin-binding motifs are highlighted in black. Conserved aromatic amino acid residues (F, Y, W) are indicated by black shading. Nearest CKI motifs are denoted in gray. All sequences were obtained from the Swiss protein database

Of note, a putative caveolin-1-binding motif, ФХФХХХХФ, is found in the sequences of other members of the TNF ligand family (Fig. 7). Thus, caveolin-1 can associate with other members of TNF ligand family, facilitating ligand translocation into rafts and triggering ligand-dependent effects.

Disruption of FasL–caveolin-1 interaction inhibits FasL translocation to rafts and FasL-induced cell death. It cannot be excluded that the functional significance of this association is much broader. The presence of several binding sites in the intracellular part of FasL, and the well-known role of caveolin as a scaffold protein, suggests the involvement of caveolin-1 in oligomerization processes within the FasL molecule^43,44^. Another possible role of this interaction is the regulation of the intracellular FasL domain translocation to the nucleus and gene transcription, respectively^45^.

In addition, this association can be involved in fulfilling the main known function of caveolin-1—suppression of malignant transformation^46^. Inability of caveolin-1 to interact with FasL, owing to mutation or phosphorylation^47,48^, may lead to the blockade of Fas-dependent cell death, preventing transformed cells’ elimination. Moreover, the ability of caveolin-1 to interact with a variety of proteins involved in signal transduction^15^ allows speculations regarding its key role in a cross-talk between different signaling pathways and FasL-dependent signaling. Future studies, including the analysis of knockout cells or animal models, in which the wild-type allele of caveolin-1 or FasL is replaced by a deletion mutant-lacking respective binding site, will help to elucidate the physiological consequences of FasL–caveolin-1 interaction.

## Materials and methods

### Antibodies and cell cultures

Mouse anti-caveolin-1 (#610406), anti-transferrin receptor (# 612124), anti-flotillin-1 (#610820), anti-Fas-ligand (#556372 clone NOK-1), anti-Fas-ligand (#556387 clone G247-4), anti-fyn (#610163), and rabbit anti-caveolin-1 (#610059) antibodies were purchased from BD Biosciences (San Diego, CA, USA). Cholera toxin, peroxidase conjugate (#227041), and mouse anti-GST antibodies (#OB03 clone 8-326) were from Calbiochem. Mouse anti-Fas antibodies (#610198) were from BD Transduction Laboratories, USA. Rabbit anti-beta-tubulin antibodies (#2146) were from Cell Signaling Technology, mouse anti-caspase 8 antibodies (#551242) were from BD Pharmingen. Purified goat anti-mouse (#170-6516) and anti-rabbit (#170-6515) IgG (H+L) horseradish peroxidase conjugates were obtained from Bio-Rad (Hercules, CA, USA). Alexa Fluor® 610-R-phycoerythrin goat anti-mouse (#A20980) and fluorescein goat anti-rabbit (#F2765) IgG (H+L) were from Invitrogen (Oregon, USA).

The following cell cultures were used: human cervical adenocarcinoma HeLa cells and african green monkey kidney cells COS-1. Сell lines were from Specialist collection of continuous cell lines of vertebrates (Institute of Cytology, Russian Academy of Sciences, St-Petersburg, Russia). Cells were maintained at 37 °C and 5% CO_2_ in DMEM (Gibco) with 10% FCS (Hyclone).

### Plasmids and recombinant proteins

The pBluescript II vector encoding full-length FasL cDNA was kindly provided by Prof. Shigekazu Nagata (Kyoto University, Japan). All FasL mutants were produced by polymerase chain reaction from a plasmid template and were cloned into the mammalian expression vector pcDNA4/TO vector (Invitrogen). To engineer the tetracycline-inducible T-REx expression of normal and mutant FasL, HeLa cells were transfected according to the manufacturer’s recommendations (Invitrogen). Caveolin-1 and DcR3 cDNAs were produced by RT-PCR with total mRNA of HeLa cells and cloned into the Signal pIg plus (Ingenius).

To produce Fc-fusion proteins, COS-1 cells were transfected with the Signal pIg plus (Ingenius) constructs containing caveolin-1, DcR3, and FasL sequences or empty vector as a control using Lipofectamin 2000™ (Invitrogen). The Fc-fusion proteins-containing incubation medium was collected, and the proteins were purified according to the manufacturer’s instructions (Ingenius).

For producing GST-fusion proteins, PCR-fragments of N- (1–101 aa) and C-terminal (135–178 aa) domains of caveolin-1 were cloned into pET42b (Novagen). Transformation of BL21(DE3) with the plasmid and recombinant protein purification were carried out according to the manufacturer’s instructions (Novagen).

For all constructs, the correct reading frames were confirmed by DNA sequencing.

For caveolin-1 knockdown, the caveolin-1-specific oligonucleotide designed according to the manufacturer’s instructions was cloned into psiRNA-h7SK (Invivogen). The caveolin-1 target sequence used is: 5′ACCTCGAGCGAGAAGCAAGTGTACGATCAAGAGTCGTACACTTGCTTCTCGCTCTT3′.

Human recombinant Fas-Ligand protein was purchased from Merck (#PF033).

### Cytotoxic assay

Cells were seeded in 96-well microtiter plates (2–3 × 10^5^ cells/ml). After treatment with tetracycline, the cells were incubated for 3–12 h. Cell viability was determined by the neutral-red uptake method^49^. The cell viability value was estimated as percentage of viable cells after treatment (untreated cells were taken as 100%).

### ^3^H-thymidine release assay

The rate of cell apoptosis was quantified as described previously^50,51^. Briefly, U937 labeled target cells (5 × 10^4^ cells/well) were coincubated with HeLa-pcDNA4/TO-FasL cells at various E:T ratios in the presence or absence of tetracycline. Data represent the mean of triplicate independent experiments. Spontaneous ^3^H-thymidine release was less than 10%. Percent cell apoptosis was calculated as [(sample cpm−spontaneous cpm)/(total cpm−spontaneous cpm)] × 100.

### Immunoblot analysis and immunofluorescent staining

Cell lysates or precipitated proteins were separated by electrophoresis through 14% SDS gels and were subsequently transferred to nitrocellulose membranes (Amersham). Blots were blocked with buffer containing 2% BSA at room temperature for 1 h and then incubated with primary antibodies against the proteins of interest overnight at room temperature. After extensive washing, primary antibodies were revealed by incubation with HRP-conjugated secondary antibodies for 1 h. Bands were visualized using ECL™ Western Blotting Analysis System and Hyperfilm™ ECL (Amersham). For double immunofluorescent staining, the HeLa cells overexpressing FasL were plated on glass coverslips and incubated in DMEM with tetracycline (1γ/ml) for 4 h, then washed twice in cold phosphate-buffered saline (PBS) and fixed by 2% paraformaldehyde. The primary antibodies were rabbit anti-caveolin-1 and murine anti-FasL. Secondary antibodies were Alexa Fluor® 610-R-phycoerythrin goat anti-mouse and fluorescein goat anti-rabbit. Three random fields per condition were acquired on the Leica confocal microscope.

### Isolation of DRMs

DRMs were isolated by using a sucrose density gradient as described previously by Brown^31^. Briefly, transfected HeLa cells (2 × 10^8^) were washed and resuspended in 1 ml of ice-cold TNET containing 25 mM Tris-Cl pH 8.0, 140 mM NaCl, 1 mM EDTA, 1% Triton X-100, supplemented with protease inhibitors cocktail (Roche). Lysates were incubated on ice at 4 °C for 30 min. The supernatants were mixed with an equal volume of 80% sucrose in TNE. A 10-ml, 5–40% gradient of sucrose in TNE buffer was layered on top of the sample, which was then centrifuged (38,000 rpm, 20 h, 4 °C) in an SW41 rotor (Beckman Coulter). Twelve 1 ml fractions were collected from the top of the gradient.

Otherwise lysates were incubated on ice at 4 °C for 30 min and then subjected to centrifugation for 5 min at 10,000 g. The supernatants were mixed with an equal volume of 80% sucrose in TNE and overlaid with 2 ml 35%, 4 ml 20%, and 2 ml 5% sucrose in TNE buffer. The gradient was centrifuged at 100,000 g for 20 h. Twenty-four 0.5 ml fractions were collected from the top of the gradient.

### Immunoprecipitation analysis

For Figs. 2a, b, d, 3c, cells were lysed in ice-cold TNET. Cell lysates were precleared with protein G-sepharose beads (GE Healthcare) and irrelevant antibodies for 1 h at 4 °C. The beads were then pelleted and precleared supernatant was incubated with specific or irrelevant antibodies overnight at 4 °C. The immune complexes were then incubated with protein G-Sepharose (25 μl) for 1 h, precipitated, and washed three times with ice-cold TNET buffer. Immunoprecipitated proteins were then analyzed by electrophoresis and immunoblotting.

For Fig. 2f, protein G-sepharose column was prepared with purified Fc-fusion proteins or recombinant Fc-fragment (as a control). Cell lysates were loaded onto the column and incubated for 16 h. After intensive washing, the bound proteins were eluted by altering the pH and analyzed by immunoblotting.

For Fig. 5b and Table 1, the recombinant full-length caveolin or its C- and N- domains were incubated with purified Fc-fusion proteins or recombinant Fc-fragment (as a control) overnight at 4 °C. The immune complexes were then incubated with protein G-Sepharose (25 μl) for 1 h, precipitated, and washed three times with ice-cold TNET buffer. Immunoprecipitated proteins were analyzed by electrophoresis and immunoblotting.
